# High school health education: The impact of medical student led instruction in northern Nevada high schools

**DOI:** 10.1016/j.pmedr.2021.101512

**Published:** 2021-08-03

**Authors:** Brandon W. Conner, Katherine G. Weller, Matt V. Biondi, Alexa R. Allen, Megan K. Rescigno, Justine L. Resnik, Sydney C. Laughton, Kendal M. Warner, Ariel E. Hierholzer, Erica Y. Kim, Molly M. Hagen, Amy A. McFarland, Reka P. Danko

**Affiliations:** a University of Nevada, Reno School of Medicine, 1664 N Virginia St, Reno, NV 89557, USA; b University of Nevada, Reno School of Community Health Sciences, 1664 N Virginia St, Reno, NV 89557, USA

**Keywords:** Adolescent behavior, Preventive health, Curricular innovation, Health education, Medical students, Patient-physician relationship

## Abstract

Health education is an important topic in high school given its lasting effect on learners. Medical students are in a unique position to deliver this curriculum as they can provide information from a relatable standpoint. Ten medical students created a health education program, The Healthier Nevada Project (HNVP), designed for high school students using four modules focused on adolescent public health concerns: substance use and addiction, exercise, personal relationships, and stress and mental health. The curriculum was administered to over 700 health class students at three schools in Reno, Nevada, U.S.A., from August 2019–March 2020. This cross-sectional study measured whether the modules increased students’ comfort level, familiarity, and likelihood of discussing each topic with a healthcare provider. The method of evaluation was pre- and post-Likert scale surveys with 7–10 questions regarding students’ understanding of each topic, knowledge of related resources, and likelihood of future discussions with healthcare providers. Linear regression analysis showed significant increases in mean scores (in all cases p < 0.0001) for all four modules after the training. The modules were adjusted for the cluster effect of School and showed no significant two-way interaction between pre- and post-survey, although overall differences between schools were present. These findings indicate that HNVP significantly increased students’ knowledge, comfort, and likelihood of discussing each topic with a healthcare professional following module administration. Future efforts will aim to evaluate the long-term impact of HNVP on student behavior and evaluate if presenter type influences program success among adolescents.

## Highlights

• Medical students teach effective school-based intervention classes.
• Program increases likelihood of discussing sensitive topics with providers.
• Substance abuse classes had the largest mean % increase in student response.
• Personal relationships as well as exercise classes improved student response.
• Stress reduction classes had the least impact on high school students.

## 1 Introduction

Health education is one of the most important subjects in high school given its broad and lasting effect on learners. In the short term, healthy students have better educational outcomes – they get better grades, have better graduation rates, and feel more connected to their peers and teachers (Basch, 2011, Forrest et al., 2013). In the long term, preventive health information has been shown to effectively address substance use and addiction (Faggiano et al., 2008), mental health problems (Feiss et al., 2019, Mendelson et al., 2010, Neil and Christensen, 2009), dating and relationships (De La Rue et al., 2017), and physical fitness (Naylor et al., 2015). Each of these topics represents a key public health concern.

School-based intervention programs are a well-studied method for improving public health outcomes. Instilling healthy attitudes and behaviors during the formative adolescent years creates lasting positive impact by influencing adult choices (Sawyer et al., 2012, Kelder et al., 1994, Singh et al., 2008). Many studies have found significant, positive benefits from school-based intervention programs. The literature describes a broad array of effective intervention types, fitting for the diversity of students, schools, and educators. The World Health Organization (WHO)^1^ guidelines for “health-promoting schools” outline a holistic approach which uses curriculum, school culture, and community input to foster an environment that promotes lifelong well-being (World Health Organization, 2014, World Health Organization, 2009, Expert Committee,1997).

The efficacy of school-based prevention is reliant on the format and delivery of the content, as well as repeated exposures over time. Each additional program adds to the accessibility of health across socioeconomic strata and provides a supportive environment to maximize the chance that these behaviors are incorporated into students’ daily lives (Langford et al., 2014, Wolfenden et al., 2017). Therefore, programs such as the one described in this study are an important method to expose students to healthcare-related topics and reinforce preventive behaviors.

Limited data currently exists comparing the impact of different educators on youth health education programs. However, medical students provide several benefits: they have firsthand knowledge of the health challenges faced in their community. They are trained in behavior-changing techniques like motivational interviewing that have been shown to have positive impacts on intervention programs (Flattum et al., 2009, Sussman et al., 2012). Finally, they can provide information from a more approachable standpoint since they are also students.

According to the 2019 Nevada High School Youth Risk Behavior Survey (YRBS): Washoe County Special Report (Diedrick et al., 2019), which included approximately 1,000 participants, 48.7% of students reported use of e-cigarettes, 37.7% reported marijuana use, and 17.6% reported use of prescription pain medications for nonmedical purposes. Stress and mental health issues were similarly concerning: 18.7% of high school students seriously considered suicide, while 40.2% of students met the clinical definition for depression. The YRBS also found that 15.3% of high school students reported being bullied on campus, 15% reported cyber-bullying, and 7.3% reported physical dating violence. Finally, this survey found that only 45.6% of high school students were physically active at least 60 min per day on five or more days during the seven days prior to answering these questions.

In this study, a team of medical students created a health education program, The Healthier Nevada Youth Educational Modules Project (HNVP), using an interactive curriculum designed for high school students. HNVP sought to promote healthy lifestyle choices using four modules: substance use and addiction, exercise, personal relationships, and stress and mental health. The study determined whether these modules improved high school students’ comfort with and knowledge of each topic, as well as their likelihood of discussing these issues with a healthcare provider.

## 2 Methods

### 2.1 Study population

During the 2019–2020 academic year, health class students in the Washoe County School District (WCSD) from grades 9–12 at three high schools received healthcare curriculum delivered by 10 medical students from the University of Nevada, Reno School of Medicine. Schools were selected based on their interest in hosting HNVP modules. Class sizes were similar between schools but represented a range of socioeconomic statuses and ethnic diversity (Table 1). Students were primarily in 9th grade, although all grades could be present. In this convenience sample, each student present in class on the day of the module was asked to participate and complete the corresponding survey.

**Table 1:** School and student demographics at the three HNVP selected schools (Data source: Public School Review 2020–2021; https://www.publicschoolreview.com/nevadav).

School/Student Characteristics	School A	School B	School C
Total enrollment (N)	1,508	1,675	1,187
Student to teacher ratio (S:T)	26:1	25:1	20:1
Graduation Rate (%)	92%	94%	86%
Nevada testing rank (lowest possible rank = 660th)	118th	69th	634th
Students eligible for free lunch (%)	23%	13%	58%
Students eligible for reduced lunch (%)	2%	2%	8%
Caucasian enrollment (%)	53%	67%	15%
Hispanic enrollment (%)	35%	19%	72%
Other race/ethnicity enrollment (%)	12%	14%	10%
Percent male	54%	52%	57%

### 2.2 HNVP development

Topics were chosen using public health data collected from the annual Nevada YRBS and Washoe County Community Health Needs Assessment (Diedrick et al., 2019). First- and second-year medical students, all of whom had a Bachelor or Master of Science degree, designed the modules to address specific health-related concerns that high school students face. Members of the team were selected based on prior teaching experience and interest in medical outreach efforts. Each module was designed to be approximately one hour long and was developed in accordance with WCSD health education objectives in the Nevada Revised Statutes (NRS 389.018). Each module covered basic physiology, community resources, and preventive healthcare recommendations. Modules were loosely based on social learning theory, where students were asked to actively model and repeat given information as well as share their personal experiences with each topic. Short, age-appropriate surveys were created to assess understanding of key module components and participant willingness/comfort in discussing this information with healthcare providers.

### 2.3 HNVP design and implementation

In this cross-sectional study, individual modules were taught over one to two days with surveys given before and after module implementation to assess module impact. Each lesson included open discussion and at least one interactive activity. All materials were approved by the WCSD and the Institutional Review Board at the University of Nevada, Reno (IRB# 1471885–1).

#### 2.3.1 Substance use and addiction

This module contained information about different types of substance use and addiction, with a focus on opioids. Students actively participated by discussing and practicing the administration of Narcan® (Naloxone) via kits provided by the medical students. The survey measured students' attitudes and beliefs about substance use as well as their familiarity with overdose treatment options.

#### 2.3.2 Exercise

Students learned the physical and psychological benefits of exercise, along with an overview of nutritional supplements and the dangers of performance-enhancing drugs. They actively participated through a gameshow-style quiz and measured their heart and respiratory rates before and after exercise. The role that medical professionals play in addressing exercise concerns was discussed.

#### 2.3.3 Personal relationships

This module focused on healthy communication, patient-physician relationships, confidentiality, and intimate partner violence. Class participation involved instructional videos and role-playing scenarios to illustrate methods for handling relationship concerns such as trust, jealousy, and possessiveness.

#### 2.3.4 Stress and mental health

Physical, emotional, and interpersonal consequences of stress were discussed in this module, as well as the impact of technology on depression and isolation. Students brainstormed ways to reduce stress and participated in a guided meditation and stretching session. They were also provided information on numerous mental health resources.

### 2.4 Assessment of effectiveness

To determine the effectiveness of each module, students completed a brief survey at two time points: prior to starting a module (“pre”) and immediately after completing a module (“post”). Each module had a unique corresponding survey with a combination of questions and statements related to the topic (Appendices A-D). Students were asked to respond using five-point Likert scales. Responses to statements were based on agreement while answers to questions were based on a number of factors such as likelihood of performing certain health behaviors, level of interest in learning, and self-assessments of current health behaviors (Appendices A-D). Personal and demographic information was not collected to maintain anonymity and minimize social-desirability bias given the sensitive nature of many survey questions.

### 2.5 Independent variables

The main independent variable of interest was the treatment variable for survey timing (before or after the training). Because personal and demographic information was not collected, individual pre- and post-surveys could not be linked. Instead, differences in survey scores from before to after the training were modeled as a binary independent variable (pre- or post-), hereafter referred to as “Survey”.

School was also tested as an independent variable in order to assess if significant differences between pre- and post-survey scores varied by school. The variable “School” was modeled as categorical with three levels (A, B, and C) representing each school.

### 2.6 Dependent variable

The dependent variable was survey “Score”: student responses to each survey question were coded numerically and then averaged to produce an overall score. The most negative traits, perceptions, and opinions were assigned a value of “1” (e.g. strongly disagree, not at all likely, or not at all important), while the most positive traits, perceptions, and opinions were assigned a value of “5” (e.g. strongly agree, very likely, or very important; see Appendices A-D).

### 2.7 Data analysis

Two linear regression models were used to assess differences in pre- and post-training scores for each module. First, the Survey variable was modeled alone to assess overall differences in scores during the two time periods. Next, the School variable and a School*Survey interaction was added to examine if differences in scores were independent of School effects. A mixed-effects linear regression was also used to test for differences in scores while accounting for lack of independence between students who attended the same school; however, because results between the fixed- and mixed-effects regressions were the same in all cases, results are presented only for the simpler fixed-effects models. In all cases, assumptions of normality for the outcome variable were tested prior to regression analysis. Statistical analyses were conducted in SAS (SAS Institute Inc., Cary, NC, USA). Significance was assessed using 95% confidence intervals, with p-values ≤ 0.05 considered sufficient to reject the null hypothesis of no pre- to post-survey difference in scores.

## 3 Results

From August 2019 to March 2020, the HNVP modules were presented to over 700 health class students, grades 9–12, in the WCSD. Approximately 700 pre- and post-surveys were obtained per module (Table 2) with scores increasing significantly towards more positive traits, perceptions, and opinions for all schools and modules following the trainings (Table 2, Table 3). Post hoc analyses showed that 95% power was achieved for α = 0.05. Discrepancies in the number of pre- and post-survey responses collected were attributed to students arriving late to class or being dismissed early for a previously arranged activity.

**Table 2:** Summary statistics for pre- and post-module survey scores among health class students at the three participating high schools, 2019–2020 academic year, Reno, NV.

		School A			School B			School C		
Module	Survey (N)	n	Mean^a^	SEM^b^	n	Mean	SEM	n	Mean	SEM
Addiction	Pre (7 4 9)	346	3.55	0.029	187	3.59	0.041	216	3.47	0.042
	Post (7 2 4)	329	4.12	0.032	175	4.15	0.050	220	3.88	0.045
Exercise	Pre (6 4 8)	339	3.74	0.028	211	3.77	0.041	98	3.58	0.048
	Post (6 3 8)	329	4.06	0.041	206	4.09	0.042	103	3.94	0.051
Relationships	Pre (6 9 1)	337	3.59	0.030	203	3.61	0.043	151	3.50	0.041
	Post (7 2 9)	380	3.91	0.029	200	3.94	0.045	149	3.76	0.049
Stress	Pre (7 6 4)	355	3.50	0.034	211	3.51	0.043	198	3.37	0.044
	Post (7 3 4)	346	3.76	0.036	204	3.81	0.047	184	3.61	0.049

a Mean of student Likert scale response scores (scored on scale of 1 to 5) for each module, stratified by school and the two survey periods.

b SEM = Standard error of mean.

**Table 3:** Linear regressions comparing pre- and post-module survey scores, based on data collected during the 2019–2020 academic year at three high schools in Reno, NV, USA, β (SE).a.

Covariate	Levels	Addiction	Exercise	Relationships	Stress
**Model 1**^b^	Constant	3.54 (3.52–3.56)^***^	3.73 (3.51–3.94)^***^	3.58 (3.55–3.60)^***^	3.47 (3.45–3.50)^***^
Survey	Pre	ref	ref	ref	ref
	Post	0.52 (0.49–0.55)^***^	0.32 (0.29–0.35)^***^	0.31 (0.28–0.34)^***^	0.27 (0.23–0.30)^***^
**Model 2**^c^	Constant	3.55 (3.52–3.58)^***^	3.74 (3.45–3.77)^***^	3.59 (3.56–3.62)^***^	3.50 (3.47–3.53)^***^
Survey	Pre	ref	ref	ref	ref
	Post	0.57 (0.52–0.62)^***^	0.32 (0.27–0.36)^***^	0.32 (0.27–0.36)^***^	0.26 (0.21–0.31)^***^
School	A	ref	ref	ref	ref
	B	0.04 (-0.07–0.09)	0.004 (-0.06–0.07)	0.02 (-0.03–0.08)	0.01 (-0.05–0.06)
	C	−0.08 (-0.13-^-^0.03)	0.04 (-0.04–0.13)^**^	−0.09 (-0.14-^-^0.03)	−0.13 (-0.19-^-^0.07)^**^
Survey*School	p-value^d^	0.0800*	0.8786	0.7011	0.8318

Notes: NV = Nevada, β = coefficient, SE β = standard error of coefficient.

*p < 0.10.

**p < 0.05.

***p < 0.0001.

a Dependent variable = Likert scale survey score (scale of 1 to 5) tested separately for the four modules using two linear regressions.

b Model 1 = overall pre- to post-score differences.

c Model 2 = pre- to post-score differences controlling for school effects.

d p-value for interaction term (all levels combined).

### 3.1 Substance use and addiction module

A total of 749 and 724 students completed the pre- and post-surveys, respectively, for the substance use and addiction module (45.8% School A, 24.6% School B, and 29.6% School C; Table 2). Question non-response was low: the mean percent of questions skipped per survey was < 1% (mean = 0.78 ± 0.84% SD, range = 0.5–1.1%).

The univariate linear regression showed that scores were higher on post-surveys compared to pre-surveys (p < 0.001; Table 3). After controlling for possible School effects, it was found that post-survey scores remained significantly higher than pre-scores (p < 0.001), School was associated with test scores (p < 0.0001), and the interaction between Survey and School was insignificant (p = 0.080; Table 3). Variation in scores was driven by School C. Students had lower scores at School C compared to Schools A and B, while scores between Schools A and B were similar (Table 2).

### 3.2 Exercise module

A total of 648 and 638 students completed the pre- and post-surveys, respectively, for the exercise module (51.9% School A, 32.4% School B, and 15.6% School C; Table 2). The mean percent of questions skipped per survey was only 0.53% (±0.76% SD, range = 0.2–1.4%).

Scores on the exercise module survey significantly increased from the pre- to post-survey (p < 0.001; Table 3). After controlling for possible School effects, post-survey scores remained significantly higher than pre-scores (p < 0.001), School was associated with test scores (p = 0.0006), and the effect of Survey was not modified by School (p = 0.8786; Table 3). Students at Schools A and B had roughly the same pre- and post-survey scores (pre-scores = 3.74 and 3.77, percent increase = 8.5 and 8.6, respectively), while students at School C showed lower pre-scores than Schools A and B but a greater percent increase in scores from pre- to post-surveys (Table 2).

### 3.3 Personal relationships module

A total of 691 and 729 students completed the pre- and post-surveys, respectively, for the personal relationships module (50.5% School A, 28.4% School B, and 21.1% School C; Table 2). Surveys were generally completed in their entirety; the mean percent of students who skipped each survey question was 1.7% (±3.7% SD, range = 1.1–2.0%).

Scores on the personal relationships module survey were significantly higher on the post-survey compared to the pre-survey (p < 0.001; Table 3). Adding School did not change this finding (Survey: p < 0.001) but showed that School was also associated with test scores (p < 0.0015) and did not act as an effect modifier (p = 0.7011; Table 3). Students at Schools A and B had higher mean scores on the personal relationships survey than students at School C (Table 2).

### 3.4 Stress and mental health module

A total of 764 and 734 students completed the pre- and post-surveys, respectively, for the stress and mental health module (46.8% School A, 27.7% School B, and 25.5% School C; Table 2). Consistent with the other modules, item-nonresponse was low (mean = 0.41 ± 0.47% SD, range = 0.2–0.9%).

Scores on the stress module survey were significantly higher on the post-survey compared to the pre-survey (p < 0.001; Table 3). Controlling for School effects did not change this finding (Survey: p < 0.001) but additionally showed a main effect of School (p < 0.0004) with no effect modification (p = 0.8318; Table 3). Students at Schools A and B had higher mean scores on the stress and mental health survey compared to students at School C (Table 2).

## 4 Discussion

School-based prevention programs are an important avenue through which adolescents learn about healthy lifestyle behaviors. The behavioral choices and patterns established during adolescence impact individuals’ current and future health status. The module-based program developed in this paper demonstrated significantly higher student understanding and comfort level with public health-relevant topics after content delivery. This study further evaluated whether medical student-led health modules impacted high school students’ likelihood of discussing these same topics with healthcare providers.

Overall, there was a statistically significant increase in student awareness of, level of comfort with, and willingness to discuss each of the four studied topics with a healthcare provider following the HNVP presentations. Results showed that the substance use and addiction module resulted in the largest pre- to post-survey score increase. In general, students rated themselves as comparatively less comfortable with this topic on the pre-survey, potentially due to the stigma surrounding substance use and addiction. Students were less likely to have received comprehensive education on this topic in previous health classes, especially regarding the opioid epidemic.

The personal relationships and exercise modules showed the second largest increases in survey scores (Table 2): the personal relationships module was second in terms of mean percent increase at Schools A and B, while the exercise module was second at School C. Adolescents require healthcare providers to establish a nonjudgmental, open, and welcoming environment before they are likely to share sensitive information such as the intimate content of the personal relationships module (American College of Obstetricians and Gynecologists, 2018). A major reason adolescents are hesitant to share intimate information with physicians is lack of awareness regarding their right to confidentiality, which was corroborated by verbal discussion during the HNVP module (Carlisle et al., 2006, Gilbert et al., 2014). The personal relationships lecture directly defined physician-patient confidentiality and how it pertains to minors navigating the healthcare system, which likely influenced its overall success.

The exercise module had the highest pre-survey score at each school, suggesting that the students already had a higher baseline level of knowledge and comfort with this topic. This could be because Nevada schools have a required physical education curriculum that provides all students with exposure to exercise-related topics, and adolescents tend to be more comfortable discussing exercise compared to the more intimate topics covered by other modules. Interestingly, the study results also showed that students at Schools A and B had roughly the same pre- and post-survey scores, while students at School C showed lower pre-scores but a greater percent score increase from pre- to post-surveys. This may reflect a decreased interest in or reduced availability of physical education classes, school-sponsored sports, or club sports for students at School C compared to the other two schools. Of note, School C was the only Title 1 school in the study with lower reported levels of academic success compared to Schools A and B.

The stress and mental health module had the smallest mean percent increase in survey scores at each school. The explanation for this was likely that the module contained three questions evaluating students’ stress levels that were not likely to change between the pre- and post-survey (see Appendices D).

By delivering relatable and interactive educational experiences, medical students help demonstrate that healthcare providers are approachable and can offer a safe, confidential space to discuss mental, physical, and emotional problems. Although not widely described in the literature, some previous efforts have been made to study this notion. The School Health Education Program (SHEP) implemented by the John A. Burns School of Medicine and the State of Hawaii Department of Education used premedical and medical students to teach six health-related topics to high school students (Wong and Naguwa, 2010). Their program provided evidence that medical students were more effective than teachers in promoting comfortability and trust. HNVP expanded on this approach to demonstrate that medical students were not only effective at teaching high school students about important health education topics, but also in increasing high school students’ comfort with and likelihood of discussing these topics with healthcare professionals. Similarly, a University of British Columbia study found that high school students who attended healthcare communication workshops hosted by medical students asked more questions and were more likely to bring up sensitive concerns at their next healthcare visit (Towle et al., 2006). These studies and the current HNVP initiative discussed in this paper demonstrate the numerous benefits of implementing medical student-led health education programs around the country.

Finally, HNVP modules increase access to preventive healthcare. Studies show that less than half of adolescents receive preventive care, and many do not visit a primary care provider (Rand and Goldstein, 2018), a trend which was also observed in the classrooms where HNVP was presented. Regular primary care visits improve health outcomes as demonstrated by markers such as decreased emergency room visits, decreased hospitalizations, decreased healthcare costs, increased rates of vaccinations, and increased well-being for specific conditions like attention-deficit hyperactivity disorder, autism spectrum disorder, and more (Rand and Goldstein, 2018, Choque Olsson et al., 2017, Rubinstein et al., 2018, Rose et al., 2019). HNVP introduces key preventive health information, provides communication strategies, and stresses the importance of seeking healthcare.

### 4.1 Limitations, future directions, and conclusions

Data showed that high school students reported healthier attitudes regarding substance use and addiction, personal relationships, exercise, and stress and mental health after the educational modules were administered. However, this project had several limitations. While survey results reflected an overall change in attitudes toward discussing these topics with healthcare providers, this study did not evaluate whether this effected actual behavioral change or whether group-level changes were consistent at the individual level considering that individual pre- and post-surveys could not be linked. Because failing to account for within-person correlation overestimates the p-value, any resulting bias from this methodological choice would be towards the null hypothesis. Given that significant results were consistently found, increased type II errors are not a concern despite the reduced precision. Moreover, variability in pre- and post- responses was low, and pre- to post-changes in students were very small compared to sample sizes. Another important limitation was the use of Likert scales for the surveys. This scale was selected because of its ease of implementation. However, the subjective options may have caused inconsistency in how students understood and responded to questions.

Additional limitations include the self-reported nature of the surveys and the variability in the delivery of content due to multiple medical students presenting the modules. Reporting bias was minimized despite the self-report design by not collecting personal data on the surveys: students could not be linked to their surveys, resulting item non-response was consistently low, and students likely felt more comfortable providing honest responses. Each class received the lectures at different times throughout the semester, which likely meant that students had different levels of baseline knowledge about certain module topics. Lastly, since school districts outside of Nevada have different curricula and may place varying levels of emphasis on substance use, personal relationships, mental health, and physical education, this limits the generalizability of this study’s results to public schools in Nevada and to school districts outside of Nevada with comparable curricula.

This study was not designed to longitudinally assess whether participation in these educational modules translated into behavioral change or different healthcare outcomes. Therefore, future directions include evaluating students at determined intervals after module completion, which would provide more evidence for the overall effectiveness of the program. While findings from this study support that medical students may successfully change the attitudes of high school students through health education efforts, a future study can specifically compare medical students to schoolteachers, school nurses, or other individuals presenting similar content. Lastly, research has shown that many unhealthy behaviors begin prior to entry into high school; therefore, it may be beneficial to present similar, age-appropriate modules to middle school students.

Future investigations are necessary in order to further evaluate the effectiveness of prevention programs run by medical students in improving high school students’ health literacy and in closing the communication gap that currently exists between adolescents and healthcare providers.

## 5 Funding support

This study was supported by a grant from the Alpha Omega Alpha (AOA) Chapter of the University of Nevada, Reno School of Medicine.

## Declaration of Competing Interest

The authors declare that they have no known competing financial interests or personal relationships that could have appeared to influence the work reported in this paper.

## Footnotes

1 **List of Text Abbreviations:** HNVP (The Healthier Nevada Youth Educational Modules Project); WHO (World Health Organization); YRBS (Youth Risk Behavior Survey); UNR Med (University of Nevada, Reno School of Medicine); WCSD (Washoe County School District); SHEP (School Health Education Program)
