# Supplementary material for: High school health education: The impact of medical student led instruction in northern Nevada high schools
Source: Prev Med Rep. 2021 Aug 3;24:101512. doi: 10.1016/j.pmedr.2021.101512 (PMC8353355; doi:10.1016/j.pmedr.2021.101512)
Supplement: Supplementary data 1 [file mmc1.docx]

# **Appendix A. Substance Use & Addiction Module Survey**

1. When I go to the doctor, I feel comfortable telling them intimate information about my substance use and/or abuse.

**Strongly Disagree Disagree Neutral Agree Strongly Agree**

**1 2 3 4 5**

1. If you were struggling with substance abuse and/or addiction, how likely would you be to go to a doctor for assistance/treatment?

**Not At All Not Very Undecided/ Somewhat Very**

**Likely Likely Neutral Likely Likely**

**1 2 3 4 5**

1. How would you rate your current understanding of substance use, substance abuse, and addiction?

**Very Poor Poor Average Good Great**

**1 2 3 4 5**

1. How interested are you in learning more about substance use, substance abuse, and addiction?

**Not At All Not Very Undecided/ Somewhat Very**

**Interested Interested Neutral Interested Interested**

**1 2 3 4 5**

1. I know what resources are available to me if I am struggling with substance abuse and/or addiction.

**Strongly Disagree Disagree Neutral Agree Strongly Agree**

**1 2 3 4 5**

1. How important is it for you to refrain from using unhealthy and/or illegal substances?

**Not At All Not Very Undecided/ Somewhat Very**

**Important Important Neutral Important Important**

**1 2 3 4 5**

1. How would you rate your current ability to avoid using unhealthy and/or illegal substances?

**Very Poor Poor Average Good Great**

**1 2 3 4 5**

1. How would you rate your current awareness and understanding of Naloxone (“Narcan”)?

**Very Poor Poor Average Good Great**

**1 2 3 4 5**

1. How would you rate your current awareness and understanding of the opioid epidemic currently facing the United States?

**Very Poor Poor Average Good Great**

**1 2 3 4 5**

# **Appendix B.** **Exercise Module Survey**

1. When I go to the doctor, I feel comfortable telling them intimate information about my exercise habits and concerns.

**Strongly Disagree Disagree Neutral Agree Strongly Agree**

**1 2 3 4 5**

1. If you were struggling with finding an exercise plan, how likely would you be to go to a doctor for assistance?

**Not At All Not Very Undecided/ Somewhat Very**

**Likely Likely Neutral Likely Likely**

**1 2 3 4 5**

1. How would you rate your current understanding of what a healthy exercise plan is?

**Very Poor Poor Average Good Great**

**1 2 3 4 5**

1. How interested are you in learning more about exercise?

**Not At All Not Very Undecided/ Somewhat Very**

**Interested Interested Neutral Interested Interested**

**1 2 3 4 5**

1. I know what resources are available to me if I am struggling to find an exercise plan that works for me.

**Strongly Disagree Disagree Neutral Agree Strongly Agree**

**1 2 3 4 5**

1. How important is it for you to have good exercise habits?

**Not At All Not Very Undecided/ Somewhat Very**

**Important Important Neutral Important Important**

**1 2 3 4 5**

1. How would you rate your physical activity level on a daily basis?

**Very Poor Poor Average Good Great**

**1 2 3 4 5**

1. How would you rate your current understanding and awareness of dietary/exercise supplements?

**Very Poor Poor Average Good Great**

**1 2 3 4 5**

1. I think that the doctor would give me good exercise counseling.

**Strongly Disagree Disagree Neutral Agree Strongly Agree**

**1 2 3 4 5**

# **Appendix C. Personal Relationships Module Survey**

1. When I go to the doctor, I feel comfortable telling them intimate information about my relationships (including friendships and romantic partners).

**Strongly Disagree         Disagree       Neutral         Agree     Strongly Agree**

**1 2 3 4   5**

1. If you were struggling with relationship troubles, how likely would you be to go to a doctor for assistance/treatment?

**Not At All         Not Very             Undecided/       Somewhat             Very**

**Likely               Likely       Neutral            Likely      Likely**

**1                           2         3             4     5**

1. How would you rate your current understanding of how to have healthy relationships?

**Very Poor        Poor       Average           Good             Great**

**1       2 3   4     5**

1. How interested are you in learning more about healthy relationships?

**Not At All           Not Very Undecided/       Somewhat                 Very**

**Interested               Interested         Neutral        Interested    Interested**

**1                             2         3               4         5**

5. I know what resources are available to me if I am struggling with negative relationships.

**Strongly Disagree        Disagree       Neutral         Agree     Strongly Agree**

**1         2           3 4     5**

6. How important is it for you to have good communication/relationship habits?

**Not At All           Not Very   Undecided/       Somewhat                 Very**

**Important               Important         Neutral        Important    Important**

**1                             2         3               4         5**

7. How would you rate your current communication/relationship habits:

a. With your friends?

**Very Poor    Poor         Average             Good             Great**

**1 2 3     4     5**

b. With your family?

**Very Poor    Poor         Average             Good             Great**

**1 2 3     4     5**

c. With your romantic partners?

**Very Poor    Poor         Average             Good             Great**

**1 2 3     4     5**

# **Appendix D. Stress and Mental Health Module Survey**

1. When I go to the doctor, I feel comfortable telling them intimate information about my mental health (including stress level, anxieties, depressive thoughts, etc.).

**Strongly Disagree         Disagree       Neutral         Agree     Strongly Agree 1 2 3 4   5**

1. If you were struggling with stress/anxiety/depression, how likely would you be to go to a doctor for assistance/treatment?

**Not At All         Not Very             Undecided/       Somewhat              Very**

**Likely               Likely       Neutral            Likely        Likely**

**1                           2        3               4       5**

3. How would you rate your current understanding of how to deal with stress & anxiety?

**Very Poor        Poor       Average           Good             Great**

**1       2 3   4     5**

4. How interested are you in learning more about stress and ways to reduce it?

**Not At All           Not Very Undecided/       Somewhat                 Very**

**Interested               Interested         Neutral         Interested    Interested**

**1                             2         3               4         5**

5. I know what resources are available to me if I am struggling with stress/anxiety.

**Strongly Disagree        Disagree       Neutral         Agree     Strongly Agree**

**1         2           3 4    5**

6. How important is it for you to have effective ways of coping with stress and anxiety?

**Not At All           Not Very   Undecided/       Somewhat                 Very**

**Important               Important         Neutral        Important    Important**

**1                             2         3               4         5**

7. How would you rate your current stress level on a daily basis?

**Always  Very   Moderately       Sometimes  Not Very**

**Stressed                 Stressed             Stressed             Stressed               Stressed**

**1                2         3             4       5**
